# The Role of Red Wood Ants (*Formica rufa* Species Group) in Central European Forest Ecosystems—A Literature Review

**DOI:** 10.3390/insects16050518

**Published:** 2025-05-13

**Authors:** Ágnes Fürjes-Mikó, Sándor Csősz, Márton József Paulin, György Csóka

**Affiliations:** 1 Department of Forest Protection, Forest Research Institute, University of Sopron, H-3232 Mátrafüred, Hungary; furjes-miko.agnes@uni-sopron.hu (Á.F.-M.); paulin.marton@uni-sopron.hu (M.J.P.); 2 HUN-REN-ELTE-MTM Integrative Ecology Research Group, Biological Institute, Eötvös Loránd University, H-1117 Budapest, Hungary; sandorcsosz2@gmail.com; 3 Department of Systematic Zoology and Ecology, Institute of Biology, ELTE-Eötvös Loránd University, H-1053 Budapest, Hungary

**Keywords:** red wood ant, forest health, predators, honeydew, ant–aphid mutualism, soil conditions

## Abstract

Red wood ants (RWA), belonging to the *Formica rufa* species group, play a crucial and fascinating role in Central Europe’s forest ecosystems. They have a high variety of effects, which they exert around their nests. Their generalist feeding on prey in the canopies of trees lowers the frequency of defoliator outbreaks, as well as increases local biodiversity. Nearly half of their diverse diet is insects, including species considered harmful by foresters. They also have a mutualistic relation with honeydew-producing aphids and planthoppers, which connection has unclear effects on the forests. The habit of RWAs building nests could also positively influence soil composition, due to its structure and high amount of organic matter, which could potentially benefit tree growth. RWAs are also known to enhance the species richness of forests by supporting various myrmecophilous species associated with them. In this study, we review the role of RWAs in forest protection, drawing on the literature focusing on Hungary and Central Europe.

## Simple Summary

Red wood ants play many different important and fascinating ecological roles in forest ecosystems. As generalist predators, they are major natural enemies of different leaf- and needle-eating insects feeding in the canopies of forest trees (both broadleaved and conifer species). Because of this, they are important contributors to the regulation of some forest pests. They maintain mutualistic interaction with aphids and scale insects. To obtain the honeydew produced by the aphids and scale insects, the ants serve as their bodyguards and protect them from their enemies. They also transport a considerable volume of organic material into the mounds they build; this elevated nutrient content can be beneficial for the trees growing in the close vicinity. Ant hills are also hot spots of biodiversity. Their colonies provide homes for many myrmecophilous (“ant-loving”) insect species. On top of this, they play an active role in the dispersal of seeds of many herbaceous plants, such as violets, speedwells, etc. This paper summarizes the abovementioned roles and many other functions of red wood ants.

## 1. Introduction

The overall health of forests is expected to decline due to global environmental conditions as influenced by climate change and biological invasions. The significance of certain abundant woodland species, such as red wood ants (abbreviated as RWAs), for restoring or maintaining forest ecosystems is little understood on a macroecological scale and should be explored to identify features that can contribute to ecosystem stability [1,2,3].

Ants are ubiquitous in terrestrial ecosystems on Earth, except for regions past the polar circles and some small islands and play dominant roles in most habitats [4]. Their dominance and essential role in providing ecosystem services are undeniable and critically important to maintaining our environmental health. These predators are expected to play a prominent role in preventing severe forest damage. In tropical forest communities, ants are responsible for removing 60% of dead insects, and their predation rate surpasses that of spiders, even though the latter are specialized predators [5].

For example, it was documented about ants foraging in grasslands that consume two hundred times their biomass, most of which consists of insects [6]. Therefore, ant activity in the functioning of forest communities is so critical that following their experimental exclusion in another study, they found no other animal group was able to remove the large quantities of insect cadavers. As a result, these unprocessed carcasses accumulate in the area, disrupting nutrient cycling [5].

In Central European forest communities, the dominant species of RWAs belong to the subgenus *Formica sensu stricto* [7]. These species prefer preying in nearby canopies, and due to their generalist habits, they can consume large numbers of herbivorous insects on forest trees. They can effectively prevent phytophagous populations from overgrowing. This is supported by the results of several studies conducted in Europe, chiefly Central and Northern Europe [8,9,10,11,12].

In this paper, our aim is to review the European literature on the role RWAs play in protecting forests, considerably broadening the scope of our two previous reviews [13,14].

## 2. Distribution and Taxonomy

RWAs, distributed in temperate and boreal forests of the northern hemisphere, count 13 species from the Palearctic region and 19 species from the Nearctic [12]. Compared to Eurasian RWAs, Nearctic species do not build anthills, and more Nearctic species inhabit shrublands or grasslands. Eurasian species prefer living in forests, except for *Formica pratensis* (Retzius, 1783), which inhabits open meadows or forest edges. Seven mound-building RWA species are known to occur in Europe: *Formica aquilonia* (Yarrow, 1955), *Formica rufa* (Linnaeus, 1761), *Formica polyctena* (Förster, 1850), *Formica pratensis* and *Formica paralugubris* (Seifert, 1996), *Formica lugubris* (Zetterstedt, 1838), and *Formica truncorum* (Fabricius, 1804) [6,15,16,17]. Four of the mentioned species (*F. rufa*, *F. polyctena*, *F. pratensis*, and *F. truncorum*) have been reported from Hungary [18].

The taxonomy of these ants has been thoroughly studied. Their first mention dates to Linnaeus (1761), and the genus has continued to capture the interest of biologists ever since. Various researchers [19,20,21,22,23,24,25,26,27] have worked on the taxonomy of the genus. Today, a total of 54 available scientific names describe the Palaearctic representatives of the *Formica rufa* group [6].

Despite the abundance of the literature, distinguishing these species is still challenging due to the following reasons: (i) the broadly overlapping ranges of character states [28], which is known from the early ages of myrmecology [29,30], (ii) the high intraspecific diversity and complicated genetic structure [15,31,32], and (iii) the high rate of hybridization demonstrated in multiple species pairs, such as in *F. aquilonia × paralugubris* [33]; *F. aquilonia × polyctena* [34], or *F. polyctena × rufa* [35,36,37,38].

Species-level identification is performed with microscopic examination based on statistically detectable differences (body shape variables, number of their hairs, etc.) [6,39]. Recently, genetic methods have also been applied [17,40]. The European taxa, although morphologically very similar, have remarkably different behavioral traits [16]; therefore, species-level identification of colonies is often easier to perform in the field based on nesting habits and behavioral observations.

It has been observed that *F. rufa* and *F. polyctena* can not only produce hybrid offspring, but allometrosis (the presence of different species within a single colony) also occurs. This phenomenon may mostly be due to the adoption of queens and the admission of orphaned, lost specimens of the other species [41]. When observing hybrid or mixed-species colonies in the field, they are intermediate regarding their behavioral patterns.

## 3. Colony Structure and Demography

Red wood ants exhibit a complex and highly organized eusocial system. Their social structure consists of queens, workers, and males. Queens are the reproductive females. Workers are sterile females and perform all colony tasks: nest maintenance, foraging, brood care, and defense. Workers measure about 5–10 mm; queens are larger, reaching up to 12 mm. Males are produced seasonally for mating and die shortly after.

Species of the *F. rufa* group live in large colonies comprising tens of thousands of workers, and they build anthills from plant debris. Within their nests, there may be one or more queens laying eggs. Colonies with multiple queens are referred to as polygynous [42,43,44]. A colony can be monodomous, meaning it inhabits a single nest, or polydomous, consisting of a network of spatially separated but socially connected nests [45,46]. Polydomous species share food and raise their young together. New colonies are typically formed through budding, where a group of workers and one or more queens leave the parent nest to start a new colony nearby.

The area and abundance of a polydomous supercolony can be quite large. For example, supercolonies of *F. lugubris* can cover up to 70 hectares and comprise at least 1200 nests [47]. *F. polyctena* also forms polydomous colonies that cover large areas, but they have smaller nests situated near larger ones connected by a network of trails. *F. pratensis*, which primarily inhabits open grasslands, pastures, or hay meadows, also forms polydomous colonies [48]. The number of queens in a polygynous wood ant nest can vary significantly, ranging from just 2–3 reproductive queens in *Formica lugubris* populations in Switzerland [31] to several hundred in *Formica aquilonia* nests in southern Finland [49].

The case of monogynous species is more difficult. In continental Europe, *F. rufa* colonies are generally characterized by a monogynous social structure [50], but when it creates supercolonies, it usually consists of no more than 11 nests [51]. *Formica lugubris* typically has a single queen per nest in Finland, whereas in some regions of Switzerland, nests often contain multiple queens [31]. So, these species are mainly monogynous, but in some cases are facultatively polygynous species.

Eurasian RWAs have a broad altitudinal and latitudinal distribution and are found in a variety of habitats and types of forests. For example, species of the *F. rufa* group can build mounds in mixed conifer forests, single-species conifer forests, and mixed conifer-hardwood forests at different elevations. Outside of Scandinavia, *F. rufa*, *F. polyctena,* and *F. pratensis* are most frequently found at elevations below 1500 m, whereas *F. aquilonia*, *F. lugubris,* and *F. paralugubris* are usually above [52].

**Figure 1:**
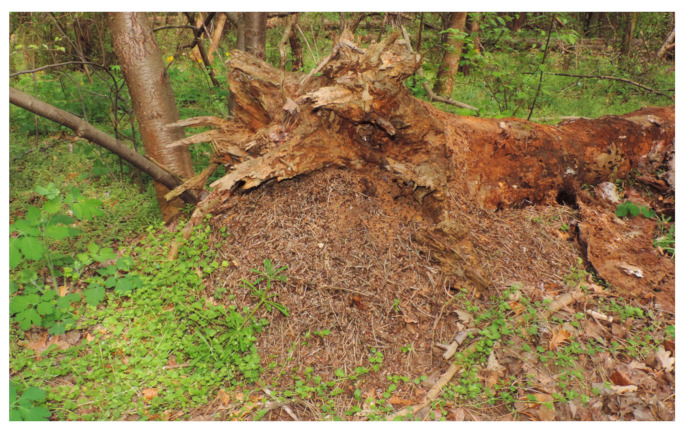
*Formica polyctena* nest on a dead pine log in a broadleaved forest (Source: György Csóka).

*F. rufa* and *F. polyctena* build their nests using plant debris and soil particles, typically on stumps or attached to deadwood (Figure 1).

Homogeneous deciduous forests are not suitable habitats for RWAs; their ecological needs are best met in well-lit mixed broadleaved-conifer forests with a south-southwest exposure [53]. One of the most important environmental factors is irradiation. It significantly influences the ants’ choice of nest sites [54], as workers often “sunbathe” outside the nest and later emit the collected heat inside [52].

Because coniferous stands are an important RWA habitat, their susceptibility to climate change is essential. In recent decades, due to deterioration, many coniferous forests have been logged. The loss of their usual habitat (e.g. clearcutting) can lead to significant changes in the ants’ nest structure, colony size, and foraging habits [55,56]. When environmental conditions become unfavorable, ants can adapt to a limit, but this often results in decreased population sizes, and this risks extinction. While the structure of RWA colonies allows them to withstand short-term impacts, they are less equipped to tolerate long-term changes [57]. However, these changes can lead to benefits. Following the large-scale bark beetle outbreak in the Białowieża Forest, researchers observed no change in the density of wood ant nests. This also suggests that although spruce is important for wood ants, when only a portion of spruce trees die, natural disturbances like bark beetle outbreaks can have beneficial effects. Increased light reaching the forest floor may promote the establishment of new nests [58].

## 4. Main Food Sources of Red Wood Ants

Most RWA species significant for forest protection are omnivorous; their diet mainly consists of insects and honeydew. They primarily consume insects in late spring and early summer; in this period, they require a large amount of protein to feed the larvae [59,60]. This is also the time during which leaf-eating organisms (caterpillars and sawfly larvae) appear in large masses.

Ants consume honeydew primarily in late summer [61] or when no other food source is available [62]. If a suitable prey insect has an outbreak, RWAs will usually stop collecting honeydew and prey on it instead [63].

According to Győrfi [64], the greatest part of their diet, 45%, consists of insects, 42% of aphid secretions (honeydew), 6% of juices exuding from plants, 4% of seeds, and 3% of fungi and other food. A study classified animals consumed by ants: 42% were considered pests by forest management, 28% were neutral, 16% were useful, while the rest (14%) could not be identified [65]. Other results showed that honeydew provides 62% of their food, insects make up 33%, and 5% is resin, fungi, carcasses, and seeds [63]. In northwest England, they observed that 70% of the ants’ nutritional needs were met by honeydew but emphasized that food composition mainly depended on the availability of food type [66]. If given the opportunity, they may also consume the juices of trees and berries [67,68].

## 5. Red Wood Ants as Biological Control Agents of Arthropods

RWAs can form supercolonies spreading large areas [12,59], prey actively in every level of the forest both during day and night, and consume any developmental stage of the insects they eat [62]. The nests of RWAs are also constant and can endure for a long time. For example, in a Swiss forest, an RWA nest has been observed for 80 years [57]. They also have direct contact with the surrounding trees and form constant systems for foraging [69].

RWAs mainly forage for food on tree trunks or in the canopy [70,71]. Their activity can form “green islands”; during the outbreak of a herbivorous insect, clumps of trees near ant nests remain green, while the rest of the forest is defoliated [72]. The abundance of leaf-eating insects is detectably decreased by the predation of ants [9,59,73,74,75].

**Figure 2:**
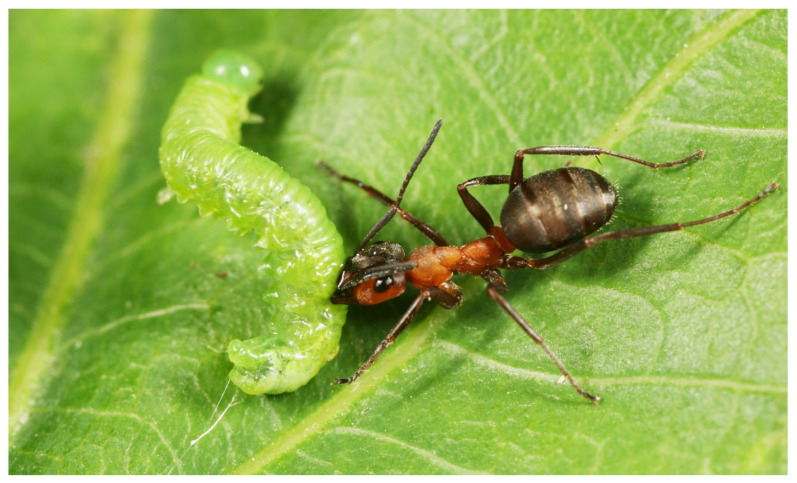
*Formica rufa* worker carrying the larva of a *Mesoneura opaca* sawfly (Source: György Csóka).

Despite the broad range of forest insects preyed on by RWAs, their effect on important insect pests is relatively understudied. Lepidopterans, flies, beetles, sawflies (Figure 2), planthoppers, bugs, bush crickets, and many other insects, as well as spiders and centipedes, are among their prey [7,64]. Information on catching beetles, however, is scarce; one report mentions that some bark beetle species are also among their prey [76].

Research data on the preying habits of RWAs is very varied [7]. In stands mixed with conifers during the outbreak of herbivores, up to 90% of the diet of *F. polyctena* may be the damaging insect [77]. A middle-sized *F. polyctena* colony can catch 8,000,000 insects within a year [63]. *F. polyctena* is also known to destroy every developmental stage of the pine beauty (*Panolis flammea*), even the pupae in the soil [5]. In the event of a pine beauty outbreak, RWAs caught 112,000 larvae in the first weeks, thus “purging” the trees near their nest of caterpillars [78,79]. In another case, when larvae of geometrid moths (Geometridae) once swarmed hawthorn bushes, *F. polyctena* established trails within a few days and almost completely freed the shrubs of the caterpillars [80].

It was recorded of an *F. rufa* colony of 200,000 workers that it collected 1000–10,000 of the small spruce sawfly (*Pristiphora abietina*) per day, and a colony of 500,000 workers up to 100,000 larvae. Once during a four-week outbreak, they were able to catch up to 1,000,000 larvae per colony, with the number depending on the size of the colony [77]. Moreover, 400 colonies can consume over one million caterpillars of the green oak roller (*Tortrix viridana*), besides other insects. Similarly, an RWA supercolony with 600 nests collected 1,000,000 winter moth (*Operophtera brumata*) and green oak roller (*Tortrix viridana*) larvae a day [81].

According to other estimates, a nest of *F. rufa* with over 100,000 workers collects nearly 60,000 insects a day [57]. It is difficult to determine the proportion of prey carried to the nest alive. In a study, 8% of the prey carried to the nest by *F. polyctena* was already dehydrated [82].

Another opinion debates the degree of generalism of RWAs (as opposed to numerous researchers) and argues that *F. rufa* picks and chooses among food sources. It prefers insects of some orders, e.g., lepidopterans (Lepidoptera) and beetles (Coleoptera), but snails (Gastropoda), spiders (Araneae), springtails (Collembola), orthopterans (Orthoptera), ants (Formicinae), and true bugs (Hemiptera) are less favored [83].

Predatory insects form a smaller part of the diet of RWAs. The most probable explanation for this is that it is more difficult to catch the usually quick predators than slower phytophagous insects [84]. It is also worth mentioning here that despite their generalist feeding, RWAs do not attack small insects. According to our own observations, they do not consume the eggs, larvae, or imagoes of, e.g., the oak lace bug (*Corythucha arcuata*), not even when they are abundant and in direct contact with the ants.

The remains of many various beetle species (e.g., Melolonthidae and Carabidae) have been found in an ant nest (*F. rufa* group), but it is uncertain whether these have been killed by the ants or have been carried to the nest already dead [65]. We observed that ants can catch and kill even small reptiles (e.g., an approximately 20 cm long blindworm (*Anguis fragilis*)).

Predation of RWAs is not limited to the daytime period, but temperature is a determining factor. Predatory activity is in linear connection with temperature and takes place between ca. 6 °C and 25 °C [85]. In the temperate zone, RWAs are inactive in winter and cool weather [7,86]. For example, in the mountainous areas of the Black Forest, a colony of *F. polyctena* did not consume the caterpillars of the European spruce budmoth (*Epinotia tedella*). These larvae are found on spruce branches at times when temperatures are too low for the ants to hunt. There was a case when RWAs did not consume the larvae of small spruce sawflies, despite their presence by the thousand near the nests [7]. The same was observed in the case of the pine looper moth (*Bupalus piniarius*): no predation took place despite the presence of *F. rufa* ants [87], although this species is normally preyed upon by every species in the *F. rufa* group [88,89,90].

Changes in the chemical composition of some plants may also cause the decrease in predation. Scots pines (*Pinus sylvestris*) contain resin acid. At high resin acid concentrations, ants are less likely to prey on insects feeding on the pine tree [91]. For better transparency, the insect species significant for forest protection and species of RWAs as their predators are listed in Table 1, along with the literature sources.

Probably one of the most important aspects of ants in forest protection may be that they keep herbivore populations at a permanently low level, reducing the risk of mass outbreaks. This effect RWA predation has on prey density (density dependence) is inadequately and probably not easily studied. But since ants are present near their hills, they constantly keep pressure even on low-density prey populations, hindering mass outbreaks. In the long run, this role may be considerable for the overall health of the forest.

RWAs can also protect forests indirectly. It was observed that near RWA nests, damage caused by the large pine weevil (*Hylobius abietis*) was smaller because ants disturbed the feeding of the weevil [116,117]. In three different Central European countries, researchers recorded that the increasing number of *F. polyctena* nests decreased damage caused by bark beetles (*Ips* spp.) [118]. The presence of ants on trees may also have a positive effect on the growth of shoots, due to the reduction in defoliation [119].

Whittaker and Warrington [120] examined the effect of the abundance of ants and leaf-eating insects on the growth of trees in the case of sycamore (*Acer pseudoplatanus*). They found that the diameter growth of trees frequented by *F. rufa* was 35–47% better than that of trees where ants were regularly present. They also found differences in the case of saplings. In all three years of the research, saplings frequented by ants experienced lower herbivore pressure, and their weight and the size of their fresh shoots were both considerably higher than those of saplings without ants.

Although they are unable to pierce the cuticle of hard, highly chitinized beetles and hairy caterpillars with their mandibles, their formic acid can kill even cockchafers [93].

Wellenstein [121] summarizes the negative and positive effects of RWAs regarding forest protection, concluding that RWAs “are considered rightfully beneficial insects that deserve protection”. This conclusion is supported by Hartner [84] as well.

## 6. Ant–Aphid Relationships

**Figure 3:**
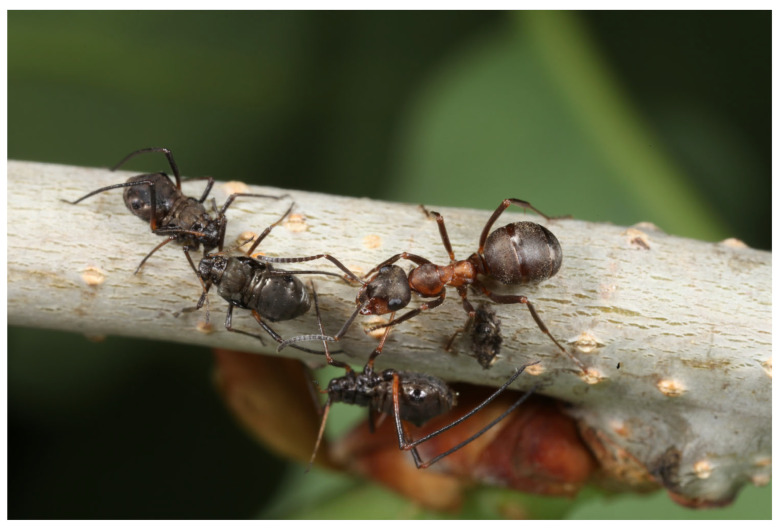
RWA and *Lachnus* aphids on oak (Source: György Csóka).

The best-known mutualistic relationship of ants is formed with Hemiptera species. As regards forest protection, the most significant relationships are those between RWAs and aphids. Ants rarely feed on these insects [66], but they regularly consume their honeydew and other secretions (Figure 3) [5]. The relationship is positive for both parties [62], as the ants obtain food while protecting the aphids from various parasitoids and predators [8,122], and even plants are protected because of the presence of ants [123]. Ants even play an important role in hygiene, as the accumulating sugary secretions of aphids would act as hotbeds for the multiplication of pathogens, for example, fungi [124]. It has only recently been discovered that members of the *F. rufa* group, *F. polyctena*, *F. pratensis*, and *F. rufa* are able to recognize aphid individuals infected by entomopathogenic fungi (*Beauveria bassiana*) and remove them instantly from the colonies, thus inhibiting the spreading of the infection. By doing so, these species actively influence the condition of the aphid colonies [125].

The result of the relationship between RWAs and aphids influencing the health and growth of trees depends on various factors, so it is not possible to give a generally valid assessment of its being either positive or negative [119,120]. Kilpeläinen et al. [126] found a statistically significant 7.3% decrease in 30-year-old stands of Norway spruce (*Picea abies*) where *F. rufa* ants and *Cinara* spp. aphids were in a mutualistic relationship. In 5-year-old stands, a non-significant increase was shown, and in older stands, no effect was observed. In the case of conifers, numerous studies have been published showing that tree growth slows down in the presence of ants [7,121,127,128,129].

The presence of RWAs can increase either the density or the productivity of aphids. For example, in the presence of *F. rufa*, the common periphyllus aphid (*Periphyllus testudinaceus*) produces several times as much honeydew as untended aphids [120]. Also, the abundance of *Symydobius oblongus* was 82 times higher on birch trees where *F. lugubris* ants were present [129]. Such occurrences may be viewed as negative for the health of the trees, but if a sufficient amount of honeydew is not available to the ants, it may diminish the size and populace of their nests [130] and the protection provided by RWAs against leaf-eating insects (as described in Section 5), and the occasional growth connected with RWA foraging [120] often compensates for the aphids’ negative effects on the plants.

## 7. Wood Ants as a Food Source for Vertebrates

It is a generally accepted fact that the majority of insectivorous songbirds do not consume RWAs. On one hand, consumption of the tiny ants is not efficient, as their nutrient and energy content is low, and on the other hand, formic acid makes them taste bad [131]. However, the role of ants is still decisive in the nutrition of some bird species. The European green woodpecker (*Picus viridis*) is one of the well-known ant specialists. Csiki [132] mentions numerous ant species, e.g., *Lasius niger*, *L. fuliginosus*, *L. flavus*, *Formica pratensis*, and *F. rubra,* from its diet. Rolstad et al. [133] demonstrated seasonal differences in the ant consumption of the green woodpecker in Scandinavia. The main food source of the birds was provided by *Serviformica* in summer and the species of the RWA in winter. Ant consumption is less typical of the grey-headed woodpecker (*Picus canus*), but when looking for food in winter, this species also often loots wood ant nests [134].

Ants are also the most important food source for the Eurasian wryneck (*Jynx torquilla*) [131,135]. The combined presence of suitable nesting places and food sources (epigeic ants) is indispensable for this species [136,137].

Among mammals, the European badger (*Meles meles*) and the wild boar (*Sus scrofa*) dig up ant nests to search for food. One of the main difficulties about protecting ant nests is, in fact, defending them against wild boar. As a curiosity, we mention that the brown bear (*Ursus arctos*) also consumes ants [138]. Its ant consumption was lower in beech forests in Slovenia than in pine forests in Sweden [139]. This is explained by the considerable differences between available ants in these two forest habitats. Bears are also known to consume ants in North America [140,141].

## 8. The Effect of Wood Ants on the Soil and Soil Fauna

Wood ants participate in the nutrient cycling of the soil. Their activity in processing organic matter enriches the soil and provides it with nutrients [142], which accumulate in high concentrations at these locations. As a result, leaf loss of the surrounding trees is reduced because their general condition is improved by utilizing these resources [9]. The chemical composition of ant nests and the surrounding soil are also different [143]. Because of the increased nutrient content and temperature inside the nest, decay processes are accelerated, and CO_2_ production is also increased. Mineralization and the accessibility of nutrients also increase [144]. In their research, however, Laakso and Setälä [145] showed that these positive effects are not prevalent outside the nests. In fact, as the ants hoard the materials responsible for better nutrient supply, the soil surrounding ant nests becomes poorer in nutrients.

*F. aquilonia* ants act negatively on the populations of epigeic insects (e.g., Carabidae) [146,147,148]. According to Duma [149], the presence of *F. rufa* has a strong effect on the invertebrate fauna of the soil. The presence of ants does not destroy soil fauna altogether but decreases its density, i.e., ants may be present in the soil parallel to other insect species. Laakso [148] observed the effects of *F. aquilonia* on endogeic animals. In the presence of ants, the species number of spiders (Araneae) and harvestmen (Opiliones) was reduced. According to Laakso and Setälä [150], ants have a beneficial effect on earthworms: their biomass was 7 times higher inside *F. aquilonia* nests. This is most probably explained by the elevated nutrient level of the nests and their surroundings. In the areas where ant nests were experimentally removed, the occurrence of the endogeic earthworm *Dendrobaena octaedra* decreased by 54% [145].

## 9. The Role of Wood Ants in Seed Dispersal

The coat of seeds collected and carried by ants is usually rich in nutrients. The seeds of some plant species contain so-called elaiosomes, “fat bodies”, which the ants can eat without damaging the germination potential of the seed. This food source may be regarded as compensation for seed dispersal undertaken by ants. This interaction is usually asymmetric, as the participating ants are generalists, i.e., not associated with single plant species [151]. This means that ants collect every seed with a portable size that they find suitable to eat. According to estimates, ants take some part in the seed dispersal of 50% of the herbaceous species of Central European broadleaved forests. According to Goesswald [89], a colony of RWAs carries and stores approximately 30,000 seeds a year. Ants “sow” the seeds of 80 plant species in oak and 45 plant species in beech forests.

Abandoned nests provide favorable conditions for seedling germination, as ant activity stops after abandonment, leading to increased moisture levels and accelerated decomposition of organic nest material. This process speeds up nutrient mineralization, making more nutrients available for plant growth [152].

## 10. Myrmecophilous Arthropods Associated with Wood Ant Colonies

Species-rich myrmecophilous (“guest of ants” or “fond of ants”) arthropod communities are associated with the colonies or nests of ants, living in special interactions with ants. Considering the species richness of myrmecophilous communities related to ant nests, the large nests of RWAs are prominent. Parmentier et al. [153] mention 125 obligate myrmecophilous species, including 52 beetles, 15 hymenopterans, 10 dipterans, 7 hemipterans, and 36 arachnids (of this, 28 are mites). Four spider species, *Mastigusa arietina*, *Thyreostenius biovatus*, *Acartauchenius scurrilis,* and *Phrurolithus festivus,* are known to occur inside mounds [154]. *Clytra* leaf beetles are typical examples of myrmecophilous beetles. The imagoes of the more common *C. laeviuscula* feed on willows; those of the rarer *C. quadripunctata* feed on oaks. The females lay their eggs near the nests or trails of ants. The eggs are carried into the nests by the ants, and, after hatching, the larvae feed on organic refuse [155,156]. The larvae form a protective mantle from their secretions and soil particles, which protects them from the ants’ mandibles.

Female copper chafers (*Potosia cuprea*) often lay their eggs near ant nests. Their larvae, accordingly, are often found in ant nests or under the heap of organic debris carried out by ants (“ant trash pile”) [157].

The family of ant-loving crickets (Orthoptera: Myrmecophilidae) is unique among orthopterans [158]. They are obligatorily connected to ants in their feeding; they cannot permanently survive without them. Their strategy can be defined as a form of cleptoparasitism. *Myrmecophilus acervorum* licks secretions from the bodies of ants and even consumes food from the mouths of ants intended for other ants. Occasionally, it also consumes ant eggs and larvae. By following the pheromone signals of the ant trails, it can transfer from one nest to the next. In Hungary, it prefers *Formica* species [159].

In northwestern and central Europe, mating adults, eggs, larvae, and pupae of *Coccinella magnifica* are associated with ants of the *F. rufa* group. This ladybug feeds on aphid species [160].

## 11. Protection of Wood Ant Species

RWA species are increasingly threatened due to two main reasons. One reason is the habitat loss due to climate change, and the other is the increasingly intensive management. In some examples, the number of RWA nests started diminishing because of intensive management practices [161,162,163,164]. Until 1994, the species group was listed in the vulnerable category on the Red List of the International Union for Conservation of Nature (IUCN); later, it was modified to near threatened. Although the *F. rufa* group is on the list, information on the species and the protective efforts aimed at them is scarce and uncoordinated. An international harmonization of the nature conservation status of RWAs would be necessary, elaborated with the participation of forest managers, as their activity greatly impacts their habitats [165].

In Hungary, the theft of ant eggs already appeared in Paragraph 258, Chapter II of Act IV of 1935: “Definition of misdemeanor in the forest”. Nowadays, Appendix 5 of Ministerial Decree No. 13/2001 (V. 9.) KöM lists protected ant species. However, only the hills of the following species are protected:

From Formica s. str.:

− Formica rufa
− Formica polyctena
− Formica truncorum
− Formica pratensis

From the Coptoformica subgenus:

− Formica pressilabris
− Formica exsecta

Effective protection of RWA nests is very difficult. RWAs provide a number of ecosystem services, which could benefit forest health, but several decades are needed for colonies to become large enough to fulfill these functions [166]. Their preservation may be mainly promoted by nature-oriented management practices. Further research addressing the protection of RWAs is also necessary. In areas where RWAs are present, but clearcutting is unavoidable, the relocation of ant nests is recommended. Recently, the nests of RWAs have been successfully relocated in European countries [167]. In Turkey, *F. rufa* is used as a means of biological control in forests, and the General Directorate of Forestry of Turkey has been relocating nests since the first half of the 20th century. Between 1941 and 2018, 12,916 nests have been relocated. Serttas et al. [168] have designed a method and software that predict the success of relocation. By supplying various habitat parameters, the environment of the present location of the nest and its intended new environment can be compared.

In the future, the continuous mapping and monitoring of ant colonies will be necessary, as well as better handling of deadwood in forests. For RWAs, deadwood is indispensable; they build their mounds on stumps and often construct their foraging trails on logs. With a suitable amount of lying deadwood available, ants build much larger nests, promoting the survival of the entire colony [169].

## 12. Summary

RWAs play a yet wholly unclarified but undeniably key role in forest ecosystems. The various services they provide also enhance forest health and promote forest protection. Their effects were at first studied in coniferous forests, but in recent decades the role of RWAs in deciduous forests has been gaining increasing attention.

RWAs are generalist predators; they forage in the canopies of trees, and their diet includes large amounts of herbivorous insects. Their feeding lowers the frequency of defoliator outbreaks and increases local biodiversity by reducing competition between herbivore species. Results quantifying herbivore reduction by RWAs are highly varied, but this is due to research conditions being highly diverse as well. Altogether, the clear conclusion is that ants can reduce the populations of defoliators.

The mutualistic relationship of RWAs and aphids is less obvious. In different forest habitats, researchers reported various effects that make it difficult to generally define whether the presence of aphids and ants together is advantageous to the trees or not. RWAs also impact seed dispersal, epigeic arthropods, soil nutrients, structure, and fauna.

There are still numerous areas that need to be researched about the effects of RWAs in forest ecosystems. However, the key services they provide for forests make it imperative that measures for their protection are implemented. Currently, their conservational status is not adequately clarified and varies by country. Because they can form large supercolonies, they might be viewed as highly resistant. Yet the growing intensity of environmental changes will cause their populations to decrease.

RWAs have a distinct connection to the forest; their impact on every level of a forest’s ecosystem makes it crucial to investigate them further, as well as include protection measures in future forest management practices.

## Figures and Tables

**Table 1:** Significant insect species in forest protection and species of RWAs that prey on them.

Order	Family	Prey	Ant Species	Tree Species	Stage	Literature
*Hymenoptera*	*Diprionidae*	*Diprion pini*	*F. polyctena*	*Pinus*	L	[65,92,93]
		*Gilpinia frutetorum*	*F. rufa group*		L	[65]
		*Gilpinia pallida*	*F. rufa group*		L	[65]
		*Neodiprion sertifer*	*F. rufa*	*Pinus*	L	[94,95]
			*F. rufa group*	*Pinus*	L	[96,97]
	*Pamphiliidae*	*Cephalcia abietis*	*F. polyctena*	*Picea*	L, I	[98]
		*Neurotoma flaviventris*	*F. rufa group*		L	[65]
	*Tenthredinidae*	*Mesoneura opaca*	*F. rufa group*	*Quercus*	L	[PD^1^]
		*Pachynematus scutellatus*	*F. rufa group*	*Larix*	L	[99]
		*Pristiphora abietina*	*F. rufa group*	*Picea*	L, P	[77,100]
			*F. polyctena*	*Picea*	L	[101]
	*Cynipidae*		*F. polyctena*		I	[PD^1^]
*Lepidoptera*	*Coleophoridae*	*Coleophora laricella*	*F. lugubris*	*Larix*	P, I	[102]
			*F. pratensis*	*Larix*	L	[103]
	*Erebidae*	*Calliteara pudibunda*	*F. polyctena*	*Fagus*	L	[104]
		*Lymantria dispar*	*F. polyctena*	*Quercus*	L	[96], [PD^1^]
			*F. rufa group*	*Quercus*	L	[104], [PD^1^]
		*Lymantria monacha*	*F. rufa group*	*Picea*, *Pinus*	P	[96,105]
			*F. polyctena*			[90]
	*Geometridae*	*Bupalus piniarius*	*F. polyctena*	*Pinus*	L, P, I	[65,88,89,90]
		*Entephria caesiata*	*F. rufa group*		L	[106]
		*Epirrita autumnata*	*F. aquilonia*	*Betula*	L	[9,59,73,76,77]
			*F. rufa group*	*Betula*	L	[107]
		*Operophtera brumata*	*F. rufa group*		L	[66,81,108], [PD^1^]
	*Lasiocampidae*	*Dendrolimus pini*	*F. rufa group*	*Pinus*	L	[89,109]
	*Noctuidae*	*Panolis flammea*	*F. polyctena*	*Pinus*	L, P, I	[5,77,78,96,104,110]
		*Conistra* sp.	*F. rufa*	*Quercus*	L	[PD^1^]
	*Notodontidae*	*Thaumetopoea pityocampa*	*F. rufa group*	*Pinus*	I	[102,111]
	*Tortricidae*	*Tortrix viridana*	*F. rufa group*	*Quercus*	L	[112,113,114,115], [PD^1^]
*Microlepidoptera*			*F. polyctena*		L	[PD^1^]
*Coleoptera*	*Curculionidae*	*Dendroctonus micans*	*F. rufa*	*Picea*	I	[76]
		*Orthotomicus erosus*				
		*Ips sexdentatus*				
		*Ips typographus*				
		*Phyllobius* sp.	*F. polyctena*	*Quercus*	I	[PD^1^]
*Hemiptera*	*Aphididae*	*Lachnus roboris*	*F. polyctena*	*Quercus*	I	[PD^1^]
*Heteroptera*	*Miridae*		*F. polyctena*		I	[PD^1^]

Abbreviations of stages: L—larva, P—pupa; I—imago. PD^1^: personal observation or unpublished data from the Chauvin trap.
